# Delay of Dental Care: An Exploratory Study of Procrastination, Dental Attendance, and Self-Reported Oral Health

**DOI:** 10.3390/dj11020056

**Published:** 2023-02-20

**Authors:** Lene M. Steinvik, Frode Svartdal, Jan-Are K. Johnsen

**Affiliations:** 1 Department of Clinical Dentistry, Faculty of Health Sciences, UiT The Arctic University of Norway, 9037 Tromsø, Norway; 2 Department of Psychology, Faculty of Health Sciences, UiT The Arctic University of Norway, 9037 Tromsø, Norway

**Keywords:** dental care, health behavior, public health dentistry, delivery of health care, procrastination, self-efficacy

## Abstract

Delay of dental care is a problem for dental public health. The present study explored the relationship between procrastination and dental attendance, focusing on delay in seeking dental care. This hypothetical relation was compared to other avoidance-related factors affecting dental attendance. In addition, an inquiry into the reasons for delaying dental care was conducted. Students (n = 164) answered an internet-based questionnaire on socio-demographic factors, dental health, dental attendance, delay of dental care, reasons for the delay, procrastination (IPS), dental anxiety (MDAS), perceived stress (PSS) and oral health self-efficacy (OHSES). The study found no significant relation between procrastination and delay in dental care. However, procrastination was related differently to past, present, and future dental attendance and seemed to relate to oral health behavior. Delay of dental care was associated with higher dental anxiety and lower oral health self-efficacy. The cost of dental care was the most frequently given reason for the delay of dental care. Further research on the delay of dental care and dental attendance is warranted in understanding the behavior, implementing interventions, and improving the utilization of public dental care.

## 1. Introduction

Dental attendance is considered an important determinant of oral health [1], and regular dental visits positively influence people’s quality of life [2]. Regular dental check-ups and scheduled appointments are preventive measures by which oral health problems and the progression of oral disease are assessed. A unique aspect of oral health is that patients are tasked with seeking professional advice preventively rather than in response to actual symptoms. This aspect is important since early-phase caries or periodontal disease can be more or less lacking in symptoms. Thus, delay of oral examinations or treatment can be assumed to affect oral health outcomes negatively. Accordingly, modern dentistry is focused on preventive measures rather than invasive treatment, which may be linked to the substantial improvements in oral health status in most Western societies during the past decades [3]. Despite prevention being a central aspect of oral health among dental health professionals, a substantial proportion of patients only seek help for acute pain or symptoms [4].

Dental anxiety or negative dental treatment experiences has often been referred to as a foundation for much avoidance behavior [5]. Studies point to the relation between dental anxiety and avoidance of dental visits, with high levels of dental anxiety predicting the length of avoidance [6]. However, delaying dental care could also be a problem among individuals that are not afraid per se but perhaps view seeking dental care as aversive or unpleasant for other reasons. For instance, patients might think that receiving treatment or undergoing dental examinations is overly expensive, time-consuming, or unnecessary due to the lack of acute symptoms [7,8]. Furthermore, it has been argued that dental attendance might be influenced by patients’ beliefs in their own ability to perform oral health behaviors. Kakudate and colleagues [9] found that individuals with lower self-efficacy were more likely to delay dental treatment. 

Procrastination is a form of self-regulatory failure that describes the failure to initiate or complete tasks [10]. Procrastination is relevant in many domains of human behavior [11]. However, it has more recently been explored in the domain of health behavior [12]. Examples of health behavior procrastination can be waiting to start or the postponement of exercise or dieting. Procrastination of health behavior is associated with less favorable health outcomes like stress and treatment delay, therefore increasing the likelihood of decreasing health status [13]. This is especially relevant for health behavior, where dental patients must initiate dental care and take charge of their own oral health and treatment. For instance, when dental patients must schedule dental appointments or when given preventive advice about the maintenance of oral hygiene, e.g., flossing. In addition, task aversiveness has been identified as a relatively strong predictor for procrastination [14], meaning that health-promoting behaviors seen as unpleasant in one way or another might be more prone to delay. Dental patients might tend to delay dental treatment for conditions that are perceived as not very serious, acute, or with low-intensity symptoms because seeking help or undergoing clinical examinations might be unpleasant in itself. 

Some evidence exists that self-regulation can affect how frequently people seek out dental treatment or examination. For instance, tendencies towards procrastination were found to be related to more infrequent visits [15]. Although seemingly trivial in some respects, delay of dental visits might have dramatic consequences, such as when patients delay seeking help for symptoms related to oral cancer [16] or neglect the progression of periodontal disease [17]. Therefore, delaying seeking dental care or avoiding treatment altogether is a problem for dental public health. Procrastination can be an important understudied construct regarding oral health behaviors, affecting the delay of dental care among patients.

The aim of this study is to explore the relationship between procrastination and dental attendance, focusing on delay in seeking dental care. In addition, the study includes an investigation of the reasons provided for delaying dental care.

## 2. Materials and Methods

Participants of the study were first-year university students at UiT The Arctic University of Norway, enrolled in health care (nursing, medicine, and dentistry), psychology, and law studies. Data were collected through convenience sampling using an internet-based questionnaire in December 2020. The questionnaire was made available to 520 students through the university’s web-based learning management system, and 195 students answered (participation rate 38%). Only 164 participants were included (participation rate 32%) due to outliers > 25 years of age. Participants were predominantly female (84.8%) with a mean age of 21 (SD = 1.96; Range 18–25 years). Responding was anonymous, and all students actively consented to participation before accessing the questionnaire. Participation was rewarded with a gift card to the local movie theatre, valued at the price of one movie ticket. Ethical and privacy concerns were addressed by the Regional Committee for Medical and Health Research Ethics (reference number 2019/572) and the Norwegian Centre for Research Data (reference number 896848). No objections were made to the study design and data collection. 

The questionnaire consisted of 20 questions regarding oral health behavior, in addition to four psychometric scales.

### 2.1. Questionnaire

Socio-demographic factors used in this study were age and gender.

Oral health was appraised by self-reported oral health and dental satisfaction. Participants were asked about how they perceive their oral health to be, using a scale from 1 (“very poor”) to 5 (“very good”), and how satisfied they were with their teeth, ranging from 1 (“very dissatisfied”) to 5 (“very satisfied”). 

Dental attendance was evaluated by questions regarding regular, past, and future dental visits. Regularity of dental visits was assessed with the question, “Do you go to the dentist/dental hygienist regularly?” where participants could respond using six response alternatives ranging from “never” to “more than once a year” (Table 1). Past dental visits were assessed with the question “When did you last go to the dentist/dental hygienist?” where participants could choose one of the four alternatives “one to six months ago” (1), “one year ago” (2), “two years ago” (3), “More than two years ago” (4). Future dental visits were assessed with the question, “Would you want to go to the dentist/dental hygienist regularly?” where participants could respond using the six response alternatives used for the regularity of dental visits.

Delay of dental care was appraised by asking, “Do you ever delay dental treatment or examinations by dentists/dental hygienists?”. Participants had five response alternatives, “never” (1), “seldom” (2), “sometimes” (3), “often” (4), and “always” (5). A dichotomized version was used when it was appropriate for the analysis using the median value. Response alternatives 1–2 indicated little or no delay, while alternatives 3–5 indicated moderate to substantial delay of dental care. 

The potential reasons for the delay were formulated as “What is the reasoning behind your postponement?” where statements related to the categories Discomfort, Stress, Fear, Cost, Time, and Necessity were given. The statements were worded as “Receiving dental treatment or examinations by dentists/dental hygienist are …”, completed with the following description “uncomfortable”, “stressful”, “scary”, “too costly”, “too time-consuming”, and “not necessary without symptoms”. All participants had to indicate to what extent the statements describe them accurately, using a scale from 1 (“does not describe me at all”), 2 (“seldom describes me”), 3 (“describes me occasionally”), 4 (“describes me often”) and 5 (“describes me well”). In the analysis, the answers indicating that the reason for the delay of dental care described the participant “often or well” (responses 4–5) were summarized, indicating how frequently the reasons were descriptive for the delay of dental care. 

Procrastination was measured using the Irrational Procrastination Scale (IPS) [18]. This is a 6-item scale where participants respond to a statement on a Likert scale from 1 to 5 in order to indicate whether the statement describes them well (5) or not (1). The statements contained descriptions of situations where postponement or delay affected the participant’s circumstances, such as “My life would be better if I did some activities or tasks earlier” or “I delay tasks beyond what is reasonable”. The scale has been validated in several European countries [19] and is used by summarizing the scores of the items. A higher score indicated procrastination tendencies. The IPS had a Cronbach’s alpha level of 0.92. 

Dental anxiety was assessed using the Modified Dental Anxiety Scale (MDAS) [20]. MDAS includes five items where participants respond using a Likert scale (1–5) and is used by summarizing the scores of the items. MDAS scores are commonly used for differentiating between low (5–10), moderate (11–18), and severe dental anxiety (>18), usually interpreted as a dental phobia (7 participants, 0.05%). The MDAS had a Cronbach’s alpha level of 0.88.

Perceived stress was measured using the Perceived Stress Scale (PSS) [21,22]. The short form PPS is a global measure of subjective stress and requires that participants evaluate four items about stressful situations in the last month on a scale from 0 (never) to 4 (very often). An example of an item is, “In the last month, how often have you felt that things were going your way?”. The PSS is used by summarizing the scores of the items. A higher score indicated more perceived stress. The PSS revealed a Cronbach’s alpha level of 0.80.

Oral health self-efficacy was appraised through the Oral Health Self-Efficacy Scale (OHSES). The OHSES was developed for this study. The development of items and factor analysis and information about the pilot study can be seen in the Supplementary Information [23]. References [23,24,25,26,27,28,29,30,31] are cited in the Supplementary Information. The scale consisted of 12 statements about the participants’ belief about their ability to take care of their oral health, perform oral hygiene, and execute dental visits, to which they agreed or disagreed on a Likert scale (1–5). All items are included in the Supplementary Information. The OHSES is used by summarizing the scores of the items. A higher score indicated more oral health self-efficacy. The OHSES had a Cronbach’s alpha of 0.81.

### 2.2. Statistical Analyses

Reliability analysis was used to investigate the internal consistency of the scales used in this study, IPS, MDAS, and PSS. A Principal Component Analysis (PCA) of the OHSES was performed as a preliminary analysis to assess the OHSES as a measurement for oral health self-efficacy. For details from this analysis, see Supporting Information.

Preliminary analyses indicated issues with the normal distribution of the dataset. Therefore, non-parametric exploratory analyses were made to investigate and explore dental attendance in the sample. The bivariate associations were assessed using Spearman’s correlation analysis. Correlations ranging from 0.2–0.4 are considered weak, 0.4–0.6 are considered moderate, and 0.6–0.8 are considered strong associations. The dichotomization of delay of dental care and OHSES based on median values was performed when appropriate for further analysis. A logistic regression analysis was performed to further explore the delay of dental care, which included significant variables from the bivariate analyses, controlling for age and gender. MDAS was dichotomized for the logistic regression analysis to indicate low (≤10) versus high levels (MDAS > 11) of dental anxiety in accordance with prior studies [32,33]. Statistical analyses were performed using IBM SPSS Statistics version 28.

## 3. Results

### 3.1. Descriptives

Demographics and background variables are shown in Table 1. With regards to oral health, 74.4% and 61.6% of participants, respectively, reported that they were of good oral health and were satisfied with their dentition. A total of 77.4% of participants had regular dental visits either every second year or more often. About eight percent (7.9%) reported attendance only with acute symptoms, and only 4.3% reported no regular attendance. Most participants (96.9%) wanted regular dental visits in the future, and 82.9% had attended dental visits during the last year. Among participants, a cumulative 39.6% reported delay in dental care sometimes, often, and always. The median and mean values of the psychometric scales in this study are shown in (Table 2). 

### 3.2. Delay of Dental Care

A correlation analysis revealed a positive correlation between age and delay of dental care (r = 0.3, *p* < 0.01), indicating that delaying dental care is more frequent among older participants in this study. Further, correlation analysis showed significant negative associations between the delay of dental care and self-reported oral health and dental satisfaction, respectively (Table 3). These associations indicated that participants with poor self-reported oral health and who indicated less dental satisfaction were also more likely to delay dental care. In order to check for the effects of dental anxiety or oral health self-efficacy on the delay of care, correlations showed that the MDAS score significantly increased with the delay of dental care (r = 0.3, *p* < 0.01), and the OHSES score significantly declined with the delay of dental care (r = −0.4, *p* < 0.01). This indicated that delay in dental care was associated with more dental anxiety and lower oral health self-efficacy. A logistic regression analysis between MDAS, OHSES, and delay of dental care, controlling for age and gender, explained 29% of the total variation in delay of dental care (Table 4). Those with moderate and severe dental anxiety had almost a three times higher probability of delaying dental care often or always (OR: 2.9, CI: 1.31–6.21). In addition, the probability of delaying dental care decreased by 78% among participants with a higher oral health self-efficacy (OR: 0.22, CI: 0.10–0.48). The results indicate that higher levels of dental anxiety and lower levels of oral health self-efficacy give an increased probability of delay in dental care.

### 3.3. Procrastination, Dental Attendance, and Self-Reported Oral Health

The correlation analysis did not reveal a significant relationship between procrastination and delay in dental care (Table 3). It was found that participants with more regular dental visits reported higher oral health self-efficacy (OHSES; r = 0.3, *p* < 0.01) and less dental anxiety (MDAS; r = −0.2, *p* < 0.01) but no significant correlation with procrastination (IPS; r = −0.2, *p* = 0.18) was found (Table 5). IPS had its strongest significant correlation with past dental visits (r = 0.2, *p* < 0.01), indicating that a greater tendency to procrastinate is associated with a prolonged time since the last dental visit. Finally, the correlation analysis revealed no significant correlation between future dental visits and the scales in this study. However, a correlation analysis was performed to further examine the relationship between procrastination and oral health. This showed a significant negative relationship (r = −0.2, *p* < 0.01), implying that low procrastination scores were related to high self-reported oral health and vice versa.

### 3.4. Reasons for Delay of Dental Care

A correlation analysis showed that the delay of dental care correlated positively and statistically significantly with all the potential reasons provided in the questionnaire (Table 3), and the strongest correlation was found between the delay of dental care and Cost (r = 0.4, *p* < 0.01). Almost half of the participants (45%) reported that the statement about perceived cost was descriptive of their delay. For the other reasons available, a smaller percentage of participants made the same judgment: Discomfort (10%), Stress (11%), Fear (7%), Time (6%), and Necessity (10%) (Table 6). MDAS and OHSES both correlated significantly but inversely with all the reasons for the delay, but the strongest correlation was found between MDAS and Discomfort, Fear, and Stress (Table 6). Procrastination (IPS) correlated significantly and positively solely with Discomfort, Stress, and Cost. 

## 4. Discussion

The current results do not support a strong relationship between procrastination and delay of dental care in this study sample. However, the results do indicate a limited association between procrastination and dental attendance. In this study, procrastination tendencies differ between past, present, and future dental attendance. There was no significant association between regular or future dental visits and procrastination but a positive association with past dental visits. In this sample, past dental visits might be a better measure of actual attendance because the data is solely based on self-report measures. Reports of, or ideas about, regular dental visits are likely to be influenced by previously learned behavior and might be affected by social norms and good intentions [34]. Thus, this could be why there was an association between past dental visits and procrastination and neither between procrastination nor hypothetical dental visits (either past or future).

As previously stated by Rhodes and Dickau [35], the problem with procrastination is not the lack of good intention but execution. Also, procrastinating might be argued to be a present-oriented act of mood regulation (through the non-execution of behavior), which is then more or less unrelated to future planning [36]. In accordance with this theoretical understanding, a large proportion of the participants in this sample expressed a desire for regular dental attendance in the future, which indicates that an intention to attend regularly is present. However, almost 40% of the participants stated that they delayed dental care to varying degrees, which points to a problem of execution related to dental visits rather than a lack of intention or future plans. Furthermore, even though this study found no significant correlation between procrastination and delay of dental care, some of the reasons for the delay are associated with procrastination. For instance, the statements regarding stress, discomfort, and cost correlated positively with procrastination tendencies, indicating that when dental treatment or examinations are perceived as stressful, unpleasant, and costly, they can be prone to procrastination driven by task aversiveness [14]. 

The most frequent response, when asked about the reason behind the delay of dental care, was the cost of dental care. This is an interesting result due to the pronominally young participants in this sample and how the Norwegian dental health services are organized. Public dental care in Norway provides free dental care to children and adolescents until 18 years of age, with additional financial support until 20 years of age [37]. After the age of 20, private dental care tends to the general adult population, where all dental care is paid for by the patient with some reimbursements related to oral diseases [37]. Thus, the sample consisted of young adults who had had regular dental care until a few years ago, with presumably good oral health, based on their self-reported oral health. Maybe the perception of the cost of dental care is a societal factor and is affected by social norms or learned behavior rather than experience. Many studies from multiple countries with diverse dental healthcare systems have shown that the cost of dental care is a common barrier to dental healthcare utilization or persistent negative perception related to dental care [38,39]. In contrast, cost perceptions of dental treatment vary greatly with regard to accuracy [40,41].

Although the delay of dental care is problematic for oral health in general, it is not the sole risk factor impacting oral health. As studied by Hagman and colleagues [42], oral health and oral health-related quality of young adults is multifactorial, affected by oral health behavior, like brushing and flossing behavior, dietary decisions, irregular dental care, and amount of gingivitis. Regarding this, procrastination could possibly be a risk factor for oral health. Previous research has demonstrated a negative relationship between overall health behaviors and procrastination [13], which indicates that procrastinators tend to practice less health-promoting behaviors. This included behaviors such as eating healthy or regular psychical exercise, which could be transferred to oral health behaviors, for instance, brushing and flossing. The current results support this assumption regarding self-reported oral health. Thus, procrastination might still be an important factor in determining oral health behavior and, ultimately, oral health status. 

While no significant relation was found between procrastination and delay of dental care, delay of dental care was associated with less oral health self-efficacy in this sample. Self-efficacy has been shown to mediate the relationship between procrastination and health behavior [13]. Sheeran and Webb [43] argue that the quality of the intention plays a substantial role in procrastination tendencies. Based on the findings in the present study, one could argue that increased oral health self-efficacy might improve the quality of the intention to seek dental care, thus decreasing the delay of dental care. Following this line of reasoning, the same could be the case with dental anxiety. In addition to good intentions, underlying knowledge about oral health is a prerequisite for the execution of health-promoting behavior [44]. The concept of oral health literacy has been suggested to mediate oral health [45] and has been linked to self-efficacy [46], dental anxiety [47], self-reported oral health [44], and dental attendance [48]. Oral health literacy was not included as a factor in the present study, which could have given further insight into the delay of dental care. 

The results showed that most participants wanted to receive regular dental care but that almost one-fourth of the participants did not attend regularly. These findings are not in line with the preventive notion of modern dentistry [3]. Previous research found few interventions targeting patients that delayed dental care and gave suggestions for the future [49]. Accordingly, summarized with our findings, interventions targeting the communication skills of oral health professionals or increasing the knowledge and understanding among patients may, in turn, increase oral health self-efficacy, decrease dental anxiety and, in turn, decrease the delay of dental care. Further investigations of motivation, intention, and cognitive aspects related to oral health behavior will be prerequisites for understanding the delay of dental care.

## 5. Strengths and Limitations

This exploratory study allows for further investigation of the behavioral patterns and cognitive aspects behind oral health behavior. While the correlations presented in this study are weak, they nonetheless give directional indications of the relationships between the variables. The most prominent limitation of this study is the sample consists of predominantly female first-year university students, making the sample a somewhat homogenous group. The overrepresentation of young and female participants may have contributed to tendencies toward healthier oral health behaviors [50], introducing statistical bias. This limits the generalization of the results to the general population. Yet there is also value in investigating this specific age group. Selecting only participants aged 18–25 gives insight into the oral health behavior of young adults. This is especially important because prevention of oral health diseases for this age group will be beneficial for their future oral health.

In addition, it can be argued that self-report measures can give rise to bias. Measures such as the past, present, and future dental attendance and oral health would be more accurate when using clinical data. Further, perhaps the measurement of stress in relation to dental visits should have been measured as acute stress rather than a steady state measurement of stress, like the perceived stress scale (PSS). Accordingly, the use of the previously unvalidated measure for oral health self-efficacy, OHSES, affects the validity of the results. The OHSES needs to be tested for reliability and validity in a larger population sample.

## 6. Conclusions

This study found no significant relation between procrastination and delay of dental care, compared to the associations found related to dental anxiety and oral health self-efficacy. However, procrastination had a different relation to past, present, and future dental attendance and could play a role in oral health behavior more specifically. Procrastination, in relation to oral health, might be more compatible with using clinical measures and actual oral health behavior. An exploration of the services provided by the Norwegian public dental services for young Norwegian adults is warranted based on these results, in particular with regard to the cost of dental care. Further research on the delay of dental care and dental attendance might be useful in understanding the behavioral patterns of patients seeking dental care, as well as implementing interventions and improving the utilization of public dental care.

## Figures and Tables

**Table 1:** Descriptive overview of demographics and variables concerning dental visits.

Variable	Category	Participants n (%)	Female n (%)	Male n (%)
**Total**		195 (100)	159 (81.5)	36 (18.5)
Age	18–25	164 (84.1)	139 (84.8)	25 (15.2)
	<25	31 (15.9)	20 (64.5)	11 (35.5)
Self-reported oral health	Very poor	0 (0)	0 (0.0)	0 (0.0)
	Poor	5 (3.0)	4 (2.9)	1 (4.0)
	Neural	36 (22.0)	29 (20.9)	7 (28.0)
	Good	90 (54.9)	77 (55.4)	13 (52.0)
	Very good	32 (19.5)	28 (20.1)	4 (16.0)
	Missing	1 (0.6)	1 (0.7)	0 (0.0)
Dental satisfaction	Very dissatisfied	2 (1.2)	2 (1.4)	0 (0.0)
	Dissatisfied	27 (16.5)	23 (16.6)	4 (16.0)
	Neural	34 (20.7)	25 (18.0)	9 (36.0)
	Satisfied	47 (28.7)	41 (29.5)	6 (24.0)
	Very satisfied	54 (32.9)	48 (34.5)	6 (24.0)
Regular dental visits	Never	7 (4.3)	4 (2.9)	3 (12.0)
	Only acute	13 (7.9)	9 (6.5)	4 (16.0)
	More seldom	17 (10.4)	14 (10.1)	3 (12.0)
	Every second year	59 (36.0)	53 (38.1)	6 (24.0)
	Once a year	63 (38.4)	54 (38.8)	9 (36.0)
	More than once a year	5 (3.0)	5 (3.6)	0 (0.0)
Future dental visits	Never	1 (0.6)	1 (0.7)	0 (0.0)
	Only acute	1 (0.6)	0 (0.0)	1 (4.0)
	More seldom	3 (1.8)	2 (1.4)	1 (4.0)
	Every second year	38 (23.2)	34 (24.5)	4 (16.0)
	Once a year	96 (58.5)	81 (58.3)	15 (60.0)
	More than once a year	25 (15.2)	21 (15.1)	4 (16.0)
Past dental visits	1–6 months	73 (44.5)	63 (45.3)	10 (40.0)
	1 year	63 (38.4)	53 (38.1)	10 (40.0)
	2 years	20 (12.2)	17 (12.2)	3 (12.0)
	Multiple years	8 (4.9)	6 (4.3)	2 (8.0)
Delay of dental care	Never	46 (28.0)	42 (30.2)	4 (16.0)
	Seldom	53 (32.4)	46 (33.1)	7 (28.0)
	Sometimes	46 (28.0)	38 (27.3)	8 (32.0)
	Often	18 (11.0)	12 (8.6)	6 (24.0)
	Always	1 (0.6)	1 (0.7)	0 (0.0)

**Table 2:** Scale properties and correlations.

Scale			Scale Correlations			
	Median (IQR)	Mean (SD)	IPS	MDAS	PSS	OHSES
IPS	19.0 (8.0)	19.4 (5.3)	1.00	0.2 *	0.4 **	−0.4 **
MDAS	9.0 (4.0)	9.9 (4.1)	0.2 *	1.00	0.2 *	−0.3 **
PSS	7.0 (3.0)	6.74 (3.2)	0.4 **	0.2 *	1.00	−0.4 **
OHSES	53.0 (10.0)	51. (6.9)	−0.4 **	−0.3 **	−0.4 **	1.00

* *p* < 0.05; ** *p* < 0.01.

**Table 3:** Spearman’s correlations between the delay of dental care, reasons for the delay, dental health and attendance, and psychometric scales.

Variables and Scales		Delay of Dental Care
Reasons for delay of dental care	Discomfort	0.3 **
	Stress	0.3 **
	Fear	0.3 **
	Cost	0.4 **
	Time	0.3 **
	Necessity	0.2 **
Oral health and dental attendance	Self-reported oral health	−0.4 **
	Dental satisfaction	−0.3 **
	Regular dental visits	−0.3 **
	Future dental visits	0.2 **
	Past dental visits	0.2 *
Psychometric scales	IPS	0.2
	MDAS	0.3 **
	PSS	0.2 *
	OHSES	−0.4 **

* *p* < 0.05; ** *p* < 0.01.

**Table 4:** Logistic regression analysis between MDAS, OHSES, and delay of dental care, controlling for age and gender.

	OR	95% CI	*p*-Value
MDAS	2.86	1.31–6.21	<0.01
OHSES	0.22	0.10–0.48	<0.01
Age	1.36	1.10–1.67	<0.01
Gender	1.93	0.63–4.93	0.21

Nagelkerke R^2^ = 0.29. OR, odds ratio; CI, confidence interval.

**Table 5:** Significant Spearman’s correlations between dental attendance variables, age, dental health, and scales.

Variables		Dental Visits		
		Regular	Past	Future
Demographics	Age	−0.3	0.10	0.1
Oral health	Self-reported oral health	0.2 *	<−0.1 *	−0.2 **
	Dental satisfaction	0.1	<0.1	−0.2 **
Psychometric scales	IPS	−0.1	0.2 **	<−0.1
	MDAS	−0.2 *	0.1	<−0.1
	PSS	−0.1	<0.1	<−0.1
	OHSES	0.2 **	−0.2 **	−0.1

* *p* < 0.05; ** *p* < 0.01.

**Table 6:** Significant Spearman’s correlations between reasons for the delay and psychometric scales and percentage of participants indicating that reasons were descriptive of their delay.

Reasons for Delay of Dental Care (%)		IPS	MDAS	OHSES	PSS
Discomfort	10%	0.2 *	0.5 **	−0.3 **	0.1
Stress	11%	0.2 *	0.6 **	−0.3 **	0.2 *
Fear	7%	0.1	0.6 **	−0.2 **	<−0.1
Cost	45%	0.2 *	0.3 **	−0.4 **	0.1
Time	6%	<0.1	0.2 *	−0.4 **	0.1
Necessity	10%	0.1	0.2 *	−0.3 **	<0.1

* *p* < 0.05; ** *p* < 0.01.

## Data Availability

Not applicable.

## Supplementary Materials

The following supporting information can be downloaded at: https://www.mdpi.com/article/10.3390/dj11020056/s1. References [23,24,25,26,27,28,29,30,31] are cited in the Supplementary Information.
