# Supplementary material for: Delay of Dental Care: An Exploratory Study of Procrastination, Dental Attendance, and Self-Reported Oral Health
Source: Dent J (Basel). 2023 Feb 20;11(2):56. doi: 10.3390/dj11020056 (PMC9955773; doi:10.3390/dj11020056)
Supplement: Supplementary file 1 [file dentistry-11-00056-s001.zip › dentistry-1897130-supplementary.pdf]

Supplementary information

The development of the Oral Health Self-Efficacy Scale (OHSES)

Previous research on self-efficacy has expressed the need for domain-specific measures, rather than general measures, for specific tasks and behavior [1]. Self-efficacy regarding oral health has previously been assessed by general measures of self-efficacy [2, 3]. While attempts have been made to produce domain-specific measures, these scales could be interpreted as either overly comprehensive [4, 5], too concise [6], or very domain-specific [7, 8]. The aim of this development was to create a domain-specific measure for oral health self-efficacy, and to explore the data created by this measure to generate future hypotheses about the underlying dimensions.

The scale items for OHSES were inspired by these previous domain-specific measures. The new items were adapted to the different aspects of oral health behaviors, and back translated from Norwegian to English. The Norwegian version was used in this study. For this paper, the items were back translated to English a second time to quality assess the translation in relation to the previous domain-specific measures of self-efficacy. The OHSES originally consisted of 14 statements about the participants' beliefs about their ability to take care of their oral health (items 1, 10, 13, and 14), to perform oral hygiene (items 2, 4, 5, 7, 9, and 11), and to execute dental visits (items 3, 6, 8, and 12), to which they agreed or disagreed on a Likert scale (1–5). The back-translated version of the scale is presented in Table S1.

**Table S1. Oral Health Self-Efficacy Scale**	
The following statements concern self-efficacy and oral health behavior. Please indicate to whether you agree or disagree with the following statements	
**Item 1**	I have belief in my own abilities to maintain good oral hygiene
**Item 2**	I find it difficult to brush my teeth
**Item 3**	It's easy for me to stick to planned dental appointments
**Item 4**	I manage to use dental floss daily
**Item 5**	I'm able to brush my teeth during times of stress or unusual situations
**Item 6**	I can book a dental appointment when I feel I need one
**Item 7**	I find it difficult to use dental floss
**Item 8**	When I'm called to make an appointment with the dentist, I make use of this
**Item 9**	I manage to brush my teeth daily
**Item 10**	I'm able to take care of my dental health in times of stress or unusual situations
**Item 11**	I'm able to use dental floss in times of stress or unusual situations
**Item 12**	I manage to go to the dentist regularly
Item 13	*I find it challenging to improve my dental health *excluded**
Item 14	*I know who to ask to answer questions I have about dental health *excluded**

Statistical analysis of the Oral Health Self-Efficacy Scale

The Oral Health Self-Efficacy Scale (OHSES) was first tested using a paper-based questionnaire among university students (n = 88) in 2019 [9]. A factor analysis was performed to determine the scale properties of the OHSES. Through a reliability analysis, the exclusion of 2 of the 14 items improved the internal consistency (Cronbach's alpha = 0.78) and face validity of the scale. The excluded items were item 13 " *I find it challenging to improve my dental health* " and item 14 "*I know who to ask to answer questions I have about dental health*". In addition, a principal component analysis revealed three underlying themes by the given three-component solution, namely oral health care and brushing behavior, dental visits, and flossing behavior. However, each component did not

independently have satisfactory internal consistency. As a conclusion to the pilot study, the OHSES was considered a holistic measure for oral health self-efficacy—with a need for further exploration and validation.

A factor analysis was performed to determine the scale properties of the OHSES in the current study (n=164). The 12 items of the OHSES were subjected to principal component analysis (PCA). Firstly, the suitability of data for factor analysis was assessed. A reliability analysis of the OHSES revealed good internal consistency (Cronbach's alpha = 0.81). Furthermore, an inspection of the correlation matrix revealed many coefficients of 0.3 and above among the items. The Kaiser–Meyer–Olkin value was 0.80, indicating sufficient data to conduct the PCA, and Bartlett's Test of Sphericity reached statistical significance at the $p < 0.001$ level. Put together, these results support the factorability of the correlation matrix. The PCA revealed three components with eigenvalues exceeding 1.0, explaining 36.7%, 12.1%, and 11.2% of the variance, respectively (Table S2). An inspection of the scree plot also indicated a three-component solution. This was further supported by the results of a parallel analysis, which showed three components with eigenvalues exceeding the corresponding criterion values compared to a randomly generated data matrix of the same size (12 items x 164) (Table S2).

Table S2. Principal component analysis (PCA) and parallel analysis

	Initial eigenvalues			Parallel analysis	
Component	Total	% of variance	Cumulative %	Random eigenvalue	Decision
1	4.403	36.690	36.690	1.4826	Accept
2	1.448	12.063	48.754	1.3521	Accept
3	1.338	11.150	59.904	1.2402	Accept
4	0.949	7.909	67.813	1.1552	Reject
5	0.830	6.920	74.732	1.0807	Reject
6	0.681	5.675	80.407	1.074	Reject
7	0.574	4.785	85.192	0.9459	Reject
8	0.485	4.044	89.236	0.8841	Reject
9	0.441	3.675	92.911	0.8185	Reject
10	0.384	3.204	96.115	0.7505	Reject
11	0.332	2.766	98.881	0.6799	Reject
12	0.134	1.119	100.000	0.6029	Reject

To further interpret these components, oblique rotation was performed. The rotated solution revealed several strong loadings in relation to the underlying construct of the items. Component 1 collected items regarding oral health care and brushing behavior (items 2, 5, 9, and 10). Component 2 collected items about dental visits (items 1, 3, 6, 8, and 12). Component 3 collected items concerning flossing behavior (items 4, 7, and 11). The component matrix, pattern matrix, and structure matrix are presented in Table S3. The internal consistency of these three components separately were acceptable with respective Cronbach's alpha values of 0.78, 0.75, and 0.73. However, the Kaiser–Meyer–Olkin values were questionable, indicating that the components were unsuited for factor analysis alone (Table S3). In addition, as seen in the structure matrix, components and items display a correlation among them, and the component matrix displayed substantial loadings for all the items on component 1. Therefore, a one-component solution appears to have some merit as a unified measure for oral health self-efficacy, yielding a total score of self-efficacy beliefs related to oral health behavior and dental visits. The one-component solution explained 36.7% of the variance in oral health self-efficacy.

Limitations

The results from this study cannot be extrapolated beyond this sample based on these findings alone. The scale has only been tested on a small Norwegian sample, and validation and investigation are warranted to assess the validity and reliability of the scale in both Norwegian and other languages. Furthermore, the scale should be compared to existing measures of self-efficacy, both general and domain-specific. The PCA contributed to the extraction of items for the scale and accounts for as much variance as possible in this sample. For future investigation, the structure of this scale would need to be cross-validated in a larger population and determine whether the common variance of each item can be explained by the underlying construct of oral health self-efficacy.

Table S3. PCA with oblique rotation

	**Component Matrix**			**Pattern Matrix**			**Structure Matrix**		
	Component			Component			Component		
	1	2	3	1	2	3	1	2	3
Item 5 MH3	0.682	-0.599	-0.052	0.972	-0.150	-0.064	0.901	0.274	0.311
Item 10 MOH4	0.717	-0.566	-0.023	0.951	-0.091	-0.007	0.910	0.301	0.364
Item 9 MH2	0.567	-0.319	-0.220	0.651	-0.183	-0.151	0.662	0.392	0.203
Item 2 MH1	0.604	-0.194	0.083	0.495	0.035	0.220	0.604	0.331	0.447
Item 3 MT1	0.601	0.316	-0.430	0.057	0.864	-0.133	0.359	0.833	0.243
Item 12 MT4	0.610	0.263	-0.252	0.079	0.630	0.090	0.380	0.700	0.381
Item 8 MT3	0.457	0.482	-0.441	-0.195	0.877	-0.059	0.144	0.772	0.214
Item 6 MT2	0.568	0.116	-0.003	0.170	0.302	0.264	0.409	0.480	0.460
Item 1 MOH1	0.672	0.120	-0.092	0.233	0.424	0.211	0.500	0.606	0.483
Item 4 MH5	0.553	0.385	0.481	-0.188	0.091	0.855	0.217	0.361	0.811
Item 11 MH6	0.689	0.138	0.396	0.149	0.042	0.712	0.472	0.394	0.793
Item 7 MH4	0.490	0.117	0.615	-0.027	-0.189	0.874	0.270	0.156	0.786
**Expected variance**	36.690	12.063	11.150	-	-	-	-	-	-
**Cronbach Alpha**	0.754	0.724	0.738	-	-	-	-	-	-
**KMO**	0.689	0.740	0.681	-	-	-	-	-	-

Rotation method: Promax with Kaiser normalization

References:

1. Leganger, A., P. Kraft, and E. Røysamb, *Perceived self-efficacy in health behaviour research: Conseptualisation, measurement and correlates.* Psychol Health, 2000. **15**(1): p. 51-69.
2. Lee, J.Y., et al., *The relationship of oral health literacy and self-efficacy with oral health status and dental neglect.* Am J Public Health, 2012. **102**(5): p. 923-9.
3. Brennan, D.S., M.M. Mittinty, and L. Jamieson, *Psychosocial factors and self-reported transitions in oral and general health.* Eur J Oral Sci, 2019. **127**(3): p. 241-247.
4. Stewart, J.E., S. Strack, and P. Graves, *Development of oral hygiene self-efficacy and outcome expectancy questionnaires.* Community Dent Oral Epidemiol, 1997. **25**(5): p. 337-42.
5. Syrjala, A.M., M.C. Kneckt, and M.L. Knuuttila, *Dental self-efficacy as a determinant to oral health behaviour, oral hygiene and HbA1c level among diabetic patients.* J Clin Periodontol, 1999. **26**(9): p. 616-21.
6. Buglar, M.E., K.M. White, and N.G. Robinson, *The role of self-efficacy in dental patients' brushing and flossing: testing an extended Health Belief Model.* Patient Educ Couns, 2010. **78**(2): p. 269-72.
7. Finlayson, T.L., et al., *Reliability and validity of brief measures of oral health-related knowledge, fatalism, and self-efficacy in mothers of African American children.* Pediatr Dent, 2005. **27**(5): p. 422-8.
8. Kakudate, N., et al., *Development of the self-efficacy scale for maternal oral care.* Pediatr Dent, 2010. **32**(4): p. 310-5.
9. Steinvik, L.M., J.-A.K. Johnsen, and F. Svartdal, *The measurement of oral health self-efficacy, and its relation to procrastination*, in *CED-IADR/NOF Oral Health Research Congress*. 2019: Madrid.
